# The Effect of Stray Current on Calcium Leaching of Cement-Based Materials

**DOI:** 10.3390/ma15062279

**Published:** 2022-03-19

**Authors:** Fang Liu, Yuanrui Zou, Baomin Wang, Xiaosa Yuan

**Affiliations:** 1 Shaanxi Key Laboratory of Safety and Durability of Concrete Structures, Xijing University, Xi’an 710123, China; liufang_winter@163.com (F.L.); zouyuanrui2021@163.com (Y.Z.); yuanxiaosa2009@163.com (X.Y.); 2 School of Civil Engineering, Dalian University of Technology, Dalian 116023, China

**Keywords:** stray current, calcium soluble corrosion, water-binder ratio, silica fume, fly ash

## Abstract

The metro engineering is in an environment rich in stray current and groundwater, which will accelerate concrete corrosion. In this study, the corrosion of cement-based materials, under stray current, consisting of direct current, was investigated, and the effects of stray current magnitude, water-binder ratio, fly ash, and silica fume on its corrosion were analyzed. The results show that, as the energisation duration of the stray current increases, the mass of the cathode side leachables increases, and the compressive strength of the cement-based materials decreases overall; at 120 d of stray current, the water-binder ratio of 0.50 shows the least reduction in strength, compared to the others; the mass of cathode side leachables decrease significantly with the increase in fly ash content; when fly ash content is 15%, the mass of cathode side leachables is the least, and the decrease in the compressive strength at 120 d of stray current is the smallest. At 10% silica fume content, the mass of the cathode side leachables is the least, and the decrease in the compressive strength of the cement-based materials at 120 d of stray current is the smallest. In general, the corrosion resistance is relatively good at 15% fly ash content and 10% silica fume content under stray current.

## 1. Introduction

The metros in Chinese cities such as Beijing, Shanghai, and Guangzhou all use direct current electric traction. At the beginning of the metro’s operation, the insulation between the running rail and track bed is good, so there is less stray current leakage from the running rail to the surrounding medium [1]. However, as the metro runs longer, the insulation of the track in the metro station and interval tunnel decreases, due to its own aging and the influence of the external environment, and the stray current leaked from the running rail to the surrounding media increases [2].

As the vast majority of the metro is located underground, concrete in metro engineering is inevitably exposed to groundwater. Long-term immersion in water with low hardness will cause a decrease in the concentration of liquid-phase lime in cement-based materials, dissolution of solid-phase lime, and decalcification transformation or decomposition of cement hydration products, resulting in increased porosity, reduced strength and durability of the slurry, and corrosion of the cement-based materials [3]. The surface of the cement-based materials with serious corrosion will be stacked very thick, affecting the surface finish of the metro lining concrete and safe operation of the metro line in severe cases.

In recent years, the research on stray current has mostly focused on the destruction of steel in concrete exposed to stray current [4,5,6,7,8,9]. Yongjing Tang et al. [10] studied the uniform corrosion and pitting corrosion of steel bars in the concrete structures of metro projects under stray current environments; Naifeng Hong et al. [11] studied the role of stray current on reinforced concrete and the form of damage to it. The mechanical properties of the steel bars were reduced, or even completely lost, due to electrolysis; in addition, the corrosion products generated by the steel bars expanded in volume, creating stresses that caused the concrete layer to crack; Xiaojun Zhou et al. [12] investigated the deterioration mechanism of concrete that is caused by the corrosion of steel bars in stray current environments. The corrosion of reinforcement causes the corrosion stress field inside the concrete. The shortest damage time of concrete caused by corrosion stress is determined not only by the concrete protective layer, concrete tensile stress, and Direct Current (DC) density of reinforcement, but also by the mechanical parameters of concrete. The thicker the concrete cover, the greater the tensile strength and longer the failure time. Therefore, they found that concrete cracked first at the thinnest protective layer of reinforced concrete that was applied with stray current.

Additionally, chloride ions, mainly from the concrete material itself and its surroundings, are also a major cause of steel corrosion [13]. Under the coupling effect with stray current, chloride ions accelerate the migration to concrete, significantly increasing the corrosion rate of the steel bars and shortening the time from the passivation of the steel bars in concrete to the cracking of the concrete protective layer [14,15]. Gang Xu et al. [16] studied the corrosion characteristics of stainless steel rebar in concrete structures, under the coupled environment of stray current and chloride salts; Qingjun Ding et al. [17] explored the effects of stray current and the limiting concentration of chloride ions on the corrosion of steel bars, and the results revealed that the limiting concentration of chloride ions decreased as the density of stray current increased.

However, concrete itself is a channel for stray current. The chemical reactions in the pore structure and solid phase part of concrete are inevitably accelerated when stray current passes through, which ultimately affects the microstructure and durability of concrete. The study on the effects of stray current on cement-based materials is, therefore, of guiding significance for metro projects.

In this study, from the perspectives of stray current magnitude, water-binder ratio, mineral admixture, etc., the dissolution of calcium ions in cement-based materials and changes in compressive strength are studied, the corrosion law of cement-based materials under stray current is analyzed, and the effects of the simultaneous presence of stray current and corrosion on rail engineering are investigated.

## 2. Experimental

### 2.1. Raw Materials

Reference cement, produced by China United Cement Corporation, Beijing, China, was used for the test. The specifications are listed in Table 1, Table 2 and Table 3.

The Class I fly ash, produced by Dalian Huaneng Power Plant, Dalian, China, with a fineness (sieve balance from 0.045 mm square hole sieve) of 3%, specific surface area of 30,741.49 cm^2^/g, water demand ratio of 92.8%, and water content of 0.72%, was used in this paper. The analysis results of its chemical composition by X-ray fluorescence spectrometer (XRF-180003040404, Shimadzu Corporation, Kyoto, Japan) are listed in Table 4.

The silica fume used here was produced by ElkemMaterialsLtd. Shanghai, China, with the average particle size of 7.76μm and specific surface area of 16,000 m^2^/kg. The chemical composition of silica fume is shown in Table 5.

China ISO standard sand, produced by ISO Standard Sand Co., Ltd., Xiamen, China, was used here. The sliced paraffin used was a white solid block produced by Sinopharm Chemical Reagent Co., Ltd., Shanghai, China, with a melting point range of 60.0–62.0 °C. Its technical conditions are in accordance with the standard Q/CYDZ 2258–2008.

### 2.2. Mix Ratio

In this paper, the effects of corrosion on specimens were investigated for different stray currents, water-binder ratios, fly ash dosing, and silica fume dosing: (1) the voltages of the stray current were 20, 40, and 60 V, respectively; (2) the water-binder ratios were 0.40, 0.50, and 0.60, respectively; (3) the dosing of fly ash and silica fume was 5%, 10%, and 15% (accounting for the total amount of cement-based materials). The specific ratios are listed in Table 6.

The mixtures, mixed according to the ratios, were placed in moulds for one day and then demoulded and placed in a standard curing room for 28 days. Afterward, the specimens were subjected to the stray current and related tests. The specimen size was 40 mm × 40 mm × 40 mm.

### 2.3. Test Design

**Figure 1:**
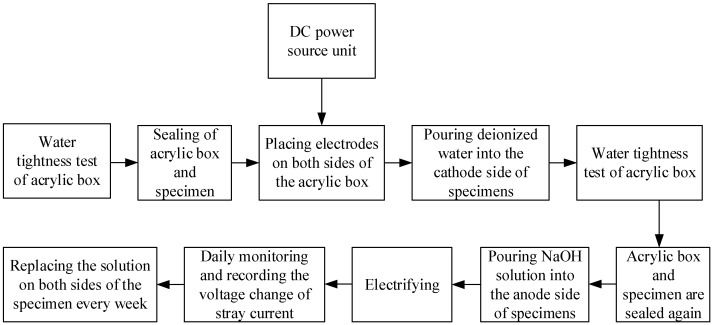
Test process of stray current.

**Figure 2:**
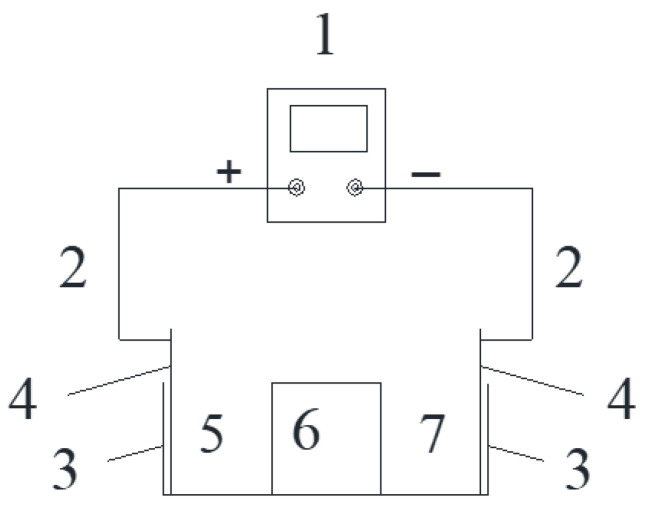
Schematic of stray current device.

**Figure 3:**
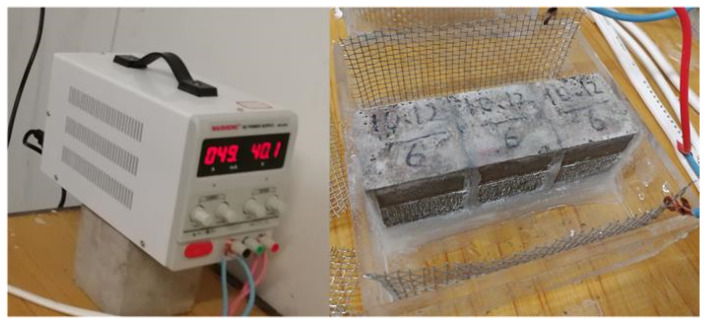
Physical map of stray current device.

After curing, the specimens (3 per group) were placed in an acrylic box (the net size of the box was 121 mm × 121 mm, height was 40 mm, and acrylic plate thickness was 5 mm), and the sides of the adjacent specimens were bonded with neutral glass adhesive. The bottom of the specimens were also coated with glass adhesive to fix them in the center of the bottom of the box, and then paraffin wax was used to cover the neutral glass adhesive and seal any gaps that may exist. The stray current test process is shown in Figure 1. The schematic diagram of the test device is shown in Figure 2. The physical stray current device is shown in Figure 3.

In this test, a stainless steel mesh was used as the electrode, the height of which was slightly higher than the inner height of the acrylic box (40 mm). The voltage of the two electrodes was measured daily with a multimeter. When the voltage was significantly lower than the test voltage, the electrodes needed to be replaced. In particular, the replacement cycle of cathode electrode should not exceed 14 days.

In the study on the corrosion of cement-based materials under stray current, the electrolysis of water by stray current normally generated H^+^ at the anode, making the anode solution acidic. Damage to cement-based materials by the acidic solution could affect the determination of the corrosion of cement-based materials under stray current. To neutralise the H^+^, a 5% NaOH solution (Tianda Chemical Reagent Factory, Tianjin, China), with the same mass fraction as the cathode side, of deionized water was placed in the electrolytic cell on the anode side. Both the 5% NaOH solution on the anode side and deionized water on the cathode side were changed every 7 days.

During the corrosion process, cathode side leachables were analyzed using an X-ray diffractometer (XRD-600003030502, Shimadzu Corporation, Kyoto, Japan, voltage/current: 40 kV/40 mA, 5.0~80.0° (2θ), step length: 0.02°, single step residence time: 0.1 s), and the compressive strength of the specimens was tested, according to the standard GB/T17671-1999 by microcomputer controlled pressure testing machine (WHY-300/10, Shanghai Hualong Testing Instrument Co., Ltd., Shanghai, China). The cathode side solution was sampled before each change of the solution of the stray current device and Ca^2+^ concentration was measured by inductively coupled plasma atomic emission spectrometry (ICP, Optima 2000DV0300429, PerkinElmer Instrument Co., Ltd., Boston, America) on a weekly basis. The effects of stray current magnitude, water-binder ratio, fly ash, and silica fume dosing on the corrosion of cement-based materials under stray current were analyzed by the above tests.

## 3. Results and Discussion

### 3.1. Effect of Stray Current on the Corrosion of Cement-Based Materials

#### 3.1.1. Cathode Side Leachables

**Figure 4:**
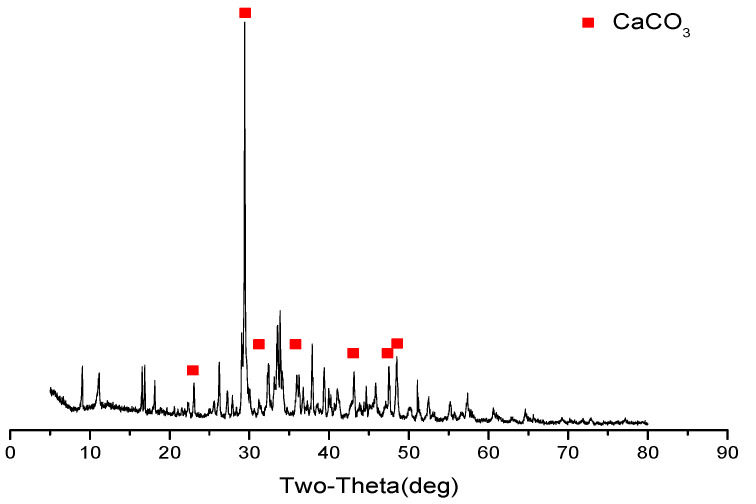
XRD analysis of cathode side leachables (mix ratio of no. 2 in Table 6, at 120 d).

When stray current is applied, white leachables on the cathode side of the specimen increase. The XRD analysis (see Figure 4) shows that the diffraction peak of CaCO_3_ is the strongest, indicating that the majority of the white leachables are CaCO_3_. Additionally, the calcium in the specimen dissolves from the cathode side, under the action of the electric field, and the aqueous solution on the cathode side becomes alkaline, under the action of electrolytic water. Ca^2+^ combines with the OH^-^ in the alkaline solution to form Ca(OH)_2_ with a relatively low solubility, which crystallized into solid Ca(OH)_2_ after reaching a certain concentration. The solid Ca(OH)_2_ tends to form CaCO_3_ with CO_2_ in the air; part of the CaCO_3_ is solidified on the cathode side of the specimen and part is retained in the cathode side solution. Therefore, when stray current is applied, white leachables can be observed on the cathode side of the specimen, and the cathode side solution also becomes cloudy with the white precipitate (CaCO_3_).

The reaction occurs on the cathode side of the stray current device, as shown in Equation (1):

(1) $$2H_{2}O+2e^{-}\;\overset{}{\to }2{\mathrm{OH}}^{-}+H_{2}$$

The reaction occurs on the anode side of the stray current device, as shown in Equation (2):

(2) $$4{\mathrm{OH}}^{-}-4e^{-}\overset{}{\to }2H_{2}O+O_{2}$$

According to Equations (1) and (2), H_2_ and O_2_ are generated on the cathode and anode sides, respectively, meaning that bubbles are generated on the two sides of the specimen. During each cycle (7 days), the frequency and number of bubbles generated and intensity of stray current are much greater on the first two days, after the solution change is completed, compared to that on the last five days, and there is white precipitation in the electrolyte solution on both sides of the specimen. It is indicated that the passing of stray current results in the formation of CaCO_3_ on the cathode side and increasing resistance of the electrolyte solution. With a constant voltage intensity, an increase in resistance results in a decrease in the intensity of stray current flowing through the specimen, and then weakens the electrolytic water reaction that occurs in the electrolyte solution on both sides of the specimen.

**Figure 5:**
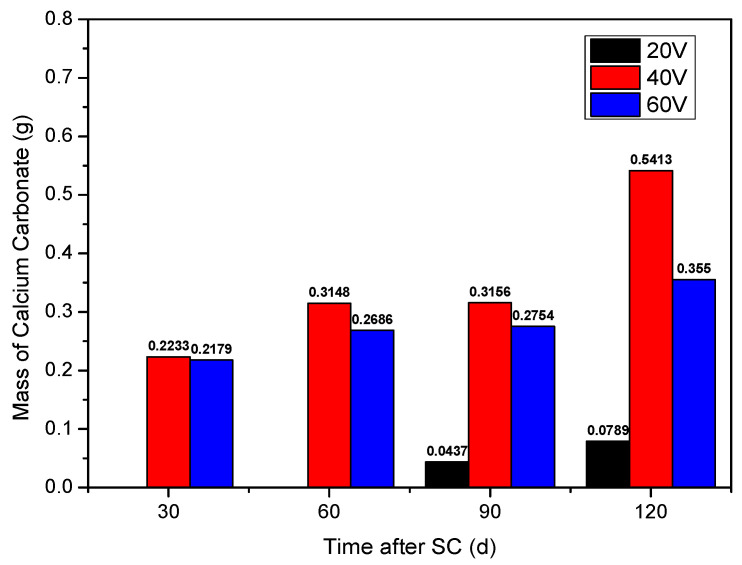
Statistics of the cathode side leachables of specimens with different stray currents.

Figure 5 shows the statistics of the cathode side leachables of specimen, with the water-binder ratio of 0.50 (mix ratio of no. 2 in Table 6), where “SC” and “d” stand for stray current and days, respectively. Leachables on the cathode side increase with the increase in the energisation duration. The accumulated mass of leachables on the cathode side of the specimen, under the voltage of 40 and 60 V, is obviously greater than that of 20 V, with a specific mass of 20 < 60 < 40 V. Leachables on the cathode side at 60 V are less than that at 40 V, which may be related to the thermal effect of stray current through cement-based materials. At the stray current voltage of 60 V, the surface temperature of cement-based material specimen is significantly higher than the body temperature, while, at the stray currents of 20 and 40 V, there is no such phenomenon. This thermal effect is equivalent of the corrosion under the coupling of stray current and temperature. The increase in the mass of cathode side leachables slows down within 60 to 90 days of electrification; after 90 days, the mass increases again significantly. When a voltage of 40 V, the CaCO_3_ dissolves on the cathode side increased by approximately 72% at 120 d of stray current, compared to that at 90 d.

#### 3.1.2. Compressive Strength

**Figure 6:**
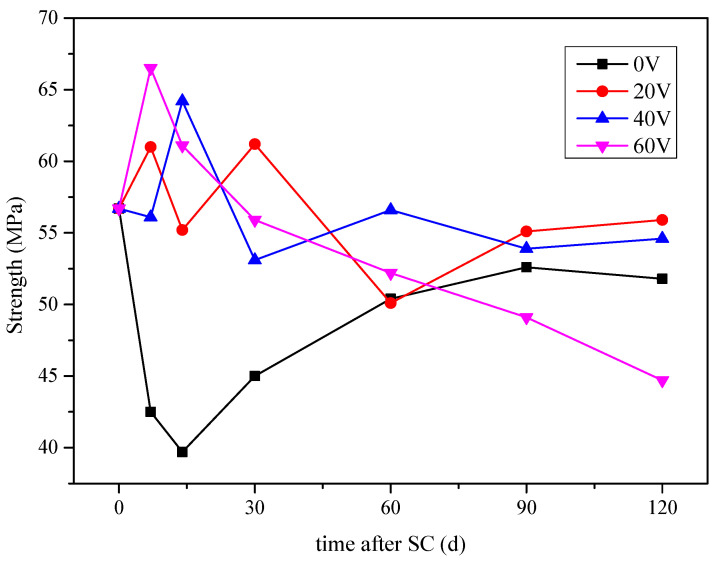
Compressive strength of the specimens under different stray currents.

The compressive strength of the specimens at 7, 14, 30, 60, 90, and 120 d of stray currents is shown in Figure 6 (mix ratio of no. 2 in Table 6). It can be concluded that there is an overall tendency for the compressive strength of cement-based materials to decrease when stray current is applied. At a voltage of 0 V, for stray current, the compressive strength of the specimen decreases considerably in the early stages of corrosion (within 14 d) and then increases, with a final decrease of 8.6% after 120 d. It may be because the 5% NaOH solution on the anode side eroded the specimen. When there is no stray current, the OH^-^ of the anode will not lose electrons, and the solution on the anode side cannot gradually change from alkaline to neutral. When the specimen is at the early stage, the strong alkaline environment may erode the specimen.

When the stray current voltage is 20 and 40 V, the cement-based material is in the environment of the coupling of stray current and corrosion. There are two factors affecting the compressive strength of cement-based materials, namely the hydration degree of cement-based materials, with the increase of age and corrosion. With the increase of hydration degree, more dense gel phases will be generated, which will enhance the compressive strength of cement-based materials. The existence of stray current will accelerate the movement of Ca^2+^ to the electrode direction. This will accelerate the dissolution of Ca(OH)_2_, or even C–S–H gel, and decrease the compressive strength of the material. Under the combined action of the two factors, the overall compressive strength of cement-based materials fluctuates and decreases with the increase of corrosion age, and tends to be stable when the age reaches 90 d.

At 120 d of stray current, the compressive strength of the specimens at voltages of 20, 40, and 60 V decreases by 1.4%, 3.7%, and 26.8%, respectively, compared to that in the pre-test period. It can be concluded that, when the voltage is 60 V, the corrosion of cement-based materials is the most serious, and the compressive strength decreases by 13.7%, compared to that of the specimen corroded with 0 V.

#### 3.1.3. Ca^2+^ Concentration in the Cathode Side Solution

**Figure 7:**
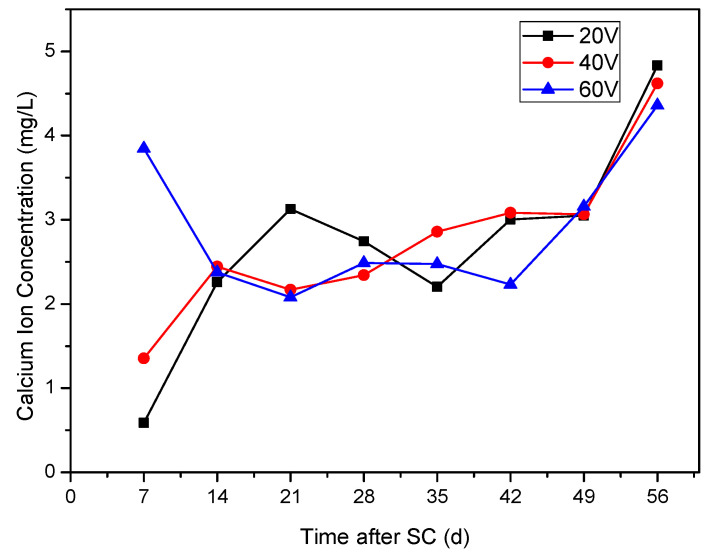
Ca^2+^ concentration in solution under different stray currents (Mix ratio of No.2 in Table 6).

As shown in Figure 7, during the first 7 days of stray current, the highest Ca^2+^ concentration in the cathode side solution is found at the voltage of 60 V, followed by the second highest Ca^2+^ concentration at 40 V, and the lowest Ca^2+^ concentration at 20 V; within 7 d to 14 d, the Ca^2+^ concentration at the voltage of 60 V decreases, while the Ca^2+^ concentration at voltages of 20 and 40 V increases; after 14 days, the Ca^2+^ concentration stabilizes at 2.2–3.2 mg/L every week. The possible reason is that, in order to maintain the internal charge balance of cement-based materials, there is a Ca^2+^ concentration threshold in the specimen. After approaching the threshold, the dissolution of Ca^2+^ slows down, so the Ca^2+^ concentration in the solution tends to be stable at 14 d. Under the voltage of 20 and 40 V, the concentration of Ca^2+^ in the solution increases continuously under the action of stray current during the age of 14 days, while, under the voltage of 60 V, the stray current accelerates the dissolution in the first 7 days, which makes the concentration of calcium ion in the solution higher. Within 7–14 days, the specimen can not continue to provide higher Ca^2+^ content, resulting in the decrease of calcium ion concentration.

After 49 d, the concentration of Ca^2+^ increases significantly, which may be because the Ca^2+^ supplied by C–H crystal inside the specimen is consumed. In order to maintain the internal charge balance of the specimen, the C–S–H gel is decomposed successively, and abundant Ca^2+^ in the specimen leads to the peak of Ca^2+^ dissolution and increase of calcium concentration in the solution.

### 3.2. Effect of Different Water-Binder Ratios on the Corrosion of Cement-Based Materials under Stray Current

#### 3.2.1. Cathode Side Leachables

**Figure 8:**
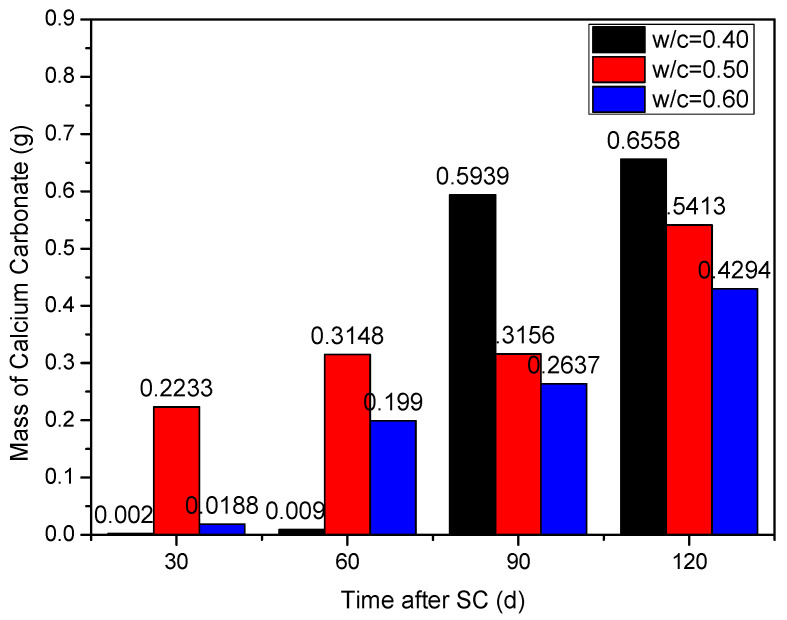
Statistics of the cathode side leachables of specimens with different water-binder ratios.

Under the action of stray current (40 V), the influence of different water-binder ratios (0.40, 0.50, and 0.60) on cathode side leachables is shown in Figure 8. It can be seen that, with the time increase, the cathode side leachables of each water-binder ratio continuously increases.

When the water-binder ratio is 0.40, there are very few leachables on the cathode side of the specimen during the first 60 d of stray current; at 90 d, the cathode side leachables increase significantly, and the cumulative mass increases by about 65.0 times, compared to that of 60 d; at 120 d, the leachables (CaCO_3_) is the most. The mass of the cathode side leachables with water-binder ratios of 0.50 and 0.60 is 17.46% and 34.52% lower, respectively, compared to those with a water-binder ratio of 0.40. In general, the corrosion resistance is the best when the water-binder ratio is 0.60. This may be because the larger the water binder ratio, the smaller the amount of cementitious material and less calcium content per unit volume. Therefore, the less cathode side leachables (CaCO_3_).

#### 3.2.2. Compressive Strength

**Figure 9:**
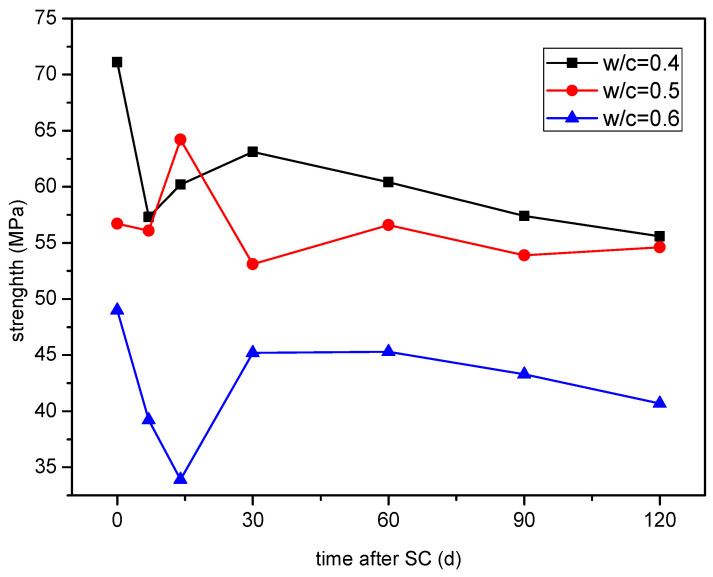
Strength of the specimens with different water-binder ratios.

The compressive strength variation of cement-based materials with different water-binder ratios under stray current is shown in Figure 9. It can be seen that the compressive strength fluctuates in the early stage, and the overall trend is decreasing in the later stage. At 120 d of stray current, the compressive strength of the specimen with a water-binder ratio of 0.50 decreases the least, by only 3.7%, while the strength of the specimens with water-binder ratios of 0.40 and 0.60 decrease by 21.8% and 16.9%, respectively.

#### 3.2.3. Ca^2+^ Concentration in the Cathode Side Solution

**Figure 10:**
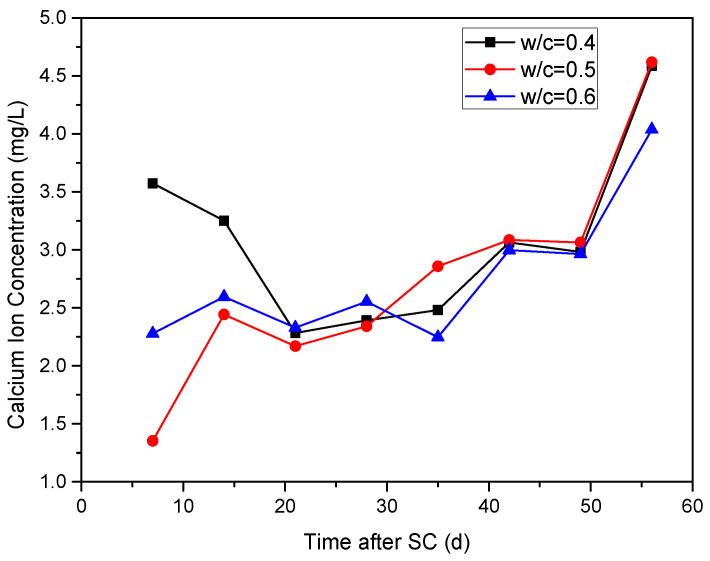
Ca^2+^ concentration in the cathode side solution of the specimens with different water-binder ratios.

The variation of Ca^2+^ concentration in the cathode side solution for specimens with different water-binder ratios under stray current is shown in Figure 10. During the first 21 d of stray current, the Ca^2+^ concentration of the cathode side solution of the specimen with a water-binder ratio of 0.40 decreases from 3.57 mg/L to 2.28 mg/L, while the Ca^2+^ concentration under water-binder ratios of 0.50 and 0.60 increase from 1.35 mg/L and 2.28 mg/L to 2.44 mg/L and 2.60 mg/L, and then drops to 2.17 mg/L and 2.33 mg/L. When stray current is applied for 21 d, the Ca^2+^ concentration in the cathode side solution of the specimens with water-binder ratios of 0.40, 0.50, and 0.60 generally shows an upward trend, indicating that the corrosion of cement-based materials is continuously intensified under stray currents. At 35 d of stray current, the Ca^2+^ concentration is the smallest when the water-binder ratio is 0.60.

### 3.3. Effect of Fly Ash on the Corrosion of Cement-Based Materials under Stray Current

#### 3.3.1. Cathode Side Leachables

**Figure 11:**
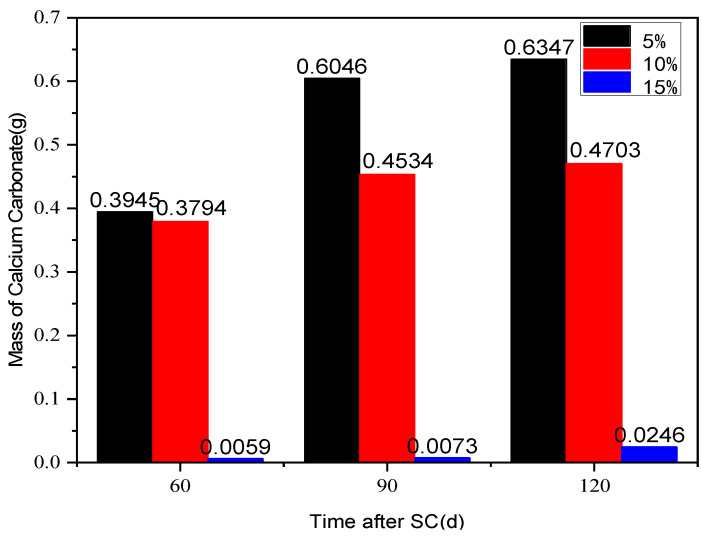
Statistics on the cathode side leachables of the specimens with different fly ash content.

The effect of different fly ash content (5%, 10% and 15%) on the corrosion of cement-based materials under stray current (40 V) at a water-binder ratio of 0.50 is shown in Figure 11. According to the figure, the mass of cathode side leachables decreases significantly with the increase of fly ash content, and the most significant effect of corrosion resistance of the specimen is observed when the fly ash content is 15%. This may be due to the different chemical composition of fly ash and cement (different calcium content). Besides, the addition of fly ash may also change the internal structure of cement-based materials. After 120 d of stray current, the cathode-side leachables of the specimens, with 5% and 10% fly ash content, increase by 24.8 and 18.1 times, respectively, compared to those with 15%.

#### 3.3.2. Compressive Strength

**Figure 12:**
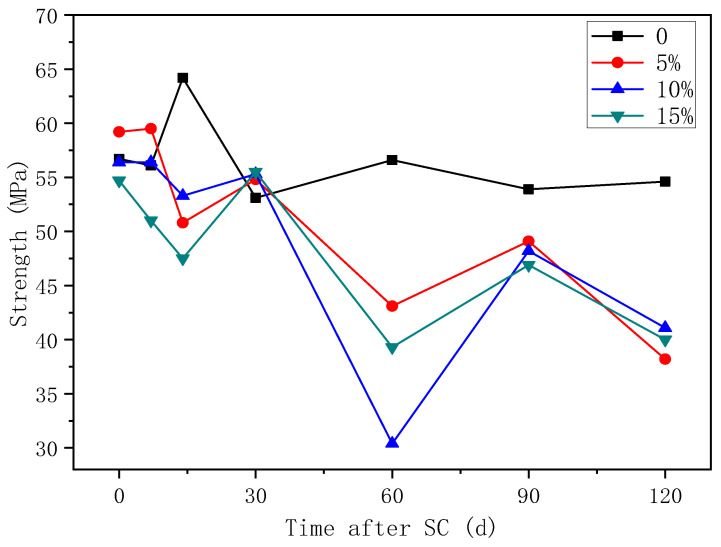
Strength of specimens with different fly ash content.

The variation of the compressive strength of cement-based materials with different fly ash content is shown in Figure 12. According to the figure, the compressive strength of the specimens tends to decrease overall as energisation duration increases, and the strength of the fly ash-doped specimen under stray current decreases more in comparison. At 120 d of stray current, the compressive strength decreases by 30.0%, 24.7%, and 26.7% at 5%, 10%, and 15% fly ash content, respectively, compared to that of the specimen without fly ash; the compressive strength decreases by 35.5%, 27.1%, and 26.9% at 5%, 10%, and 15% fly ash content, respectively, compared to the respective initial compressive strength without electricity. Therefore, the decrease in the compressive strength is the smallest at 15% fly ash content.

#### 3.3.3. Ca^2+^ Concentration in the Cathode Side Solution

**Figure 13:**
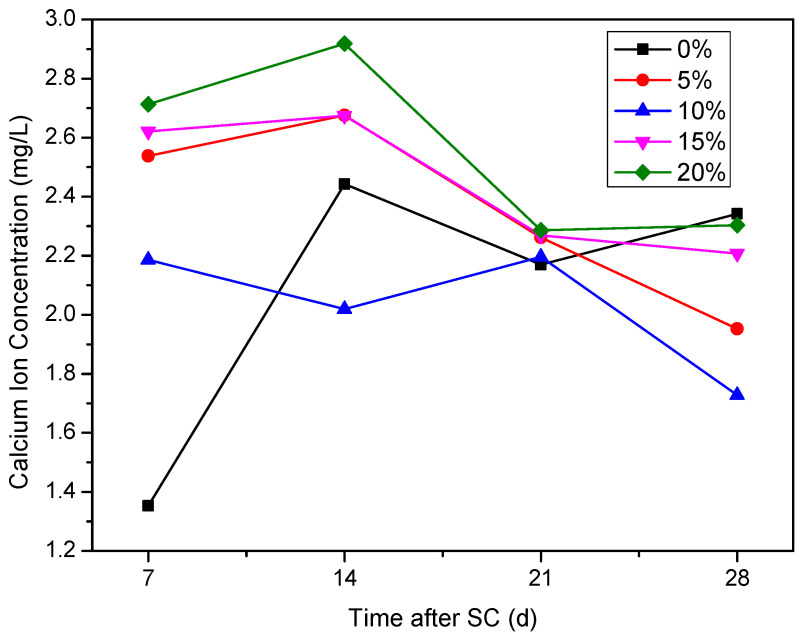
Ca^2+^ concentration on the cathode side of specimens with different fly ash content.

As can be seen from Figure 13, the Ca^2+^ concentration in the cathode side solution fluctuates with increasing stray current duration. The Ca^2+^ concentration in the cathode side solution of the specimen without fly ash is stable at 2.10 mg/L–2.45 mg/L, during 14 to 28 d of stray current. The Ca^2+^ concentration in the cathode side solution of the specimens increases during the first 14 d of stray current, when the fly ash is mixed at 5%, 15%, and 20%, indicating that the corrosion of the specimens increases under stray current; within 14 d to 28 d, the Ca^2+^ concentration in the cathode side solution of the specimens with 5%, 15%, and 20% fly ash content decreases overall, indicating that the corrosion reaction is weakened. When the content of fly ash is 10%, the Ca^2+^ concentration in the solution is generally low after 7 days of stray current; compared with other fly ash contents at 28 d of stray currents, the Ca^2+^ concentration is the lowest when the fly ash content is 10%. In general, after 21 d of stray current, the Ca^2+^ concentration of the specimen without fly ash increases, while the Ca^2+^ concentration of the specimens with fly ash decreases overall, and all are lower than those without fly ash.

### 3.4. Effect of Silica Fume on the Corrosion of Cement-Based Materials under Stray Current

#### 3.4.1. Cathode Side Leachables

**Figure 14:**
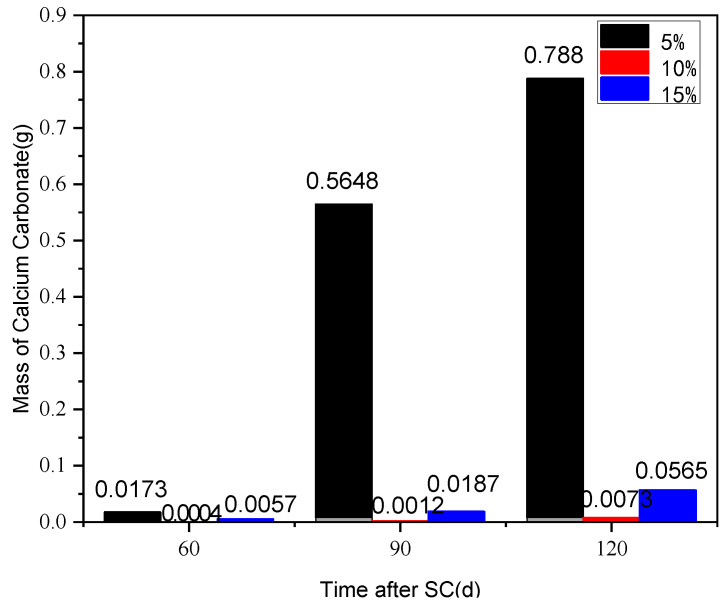
Statistics on cathode side leachables of specimens with different silica fume content.

The effect of different silica fume content (5%, 10%, and 15%) on the corrosion of cement-based materials under stray current (40 V) at a water-binder ratio of 0.50 is shown in Figure 14. According to Figure 14, when the content of silica fume is 5%, the mass of the cathode side leachables of the specimen, at 90 d of stray current, increases sharply by 31.6 times, compared with that at 60 d; with the increase in the silica fume content, the mass of cathode side leachables decreases significantly, and then slightly increases; when the silica fume content is 10%, the mass of the cathodic side leachables is minimal, in comparison. At 120 d of stray current, the cathode side leachables of the specimens with 5% and 15% silica fume increase by 106.9 and 6.7 times, respectively, compared to those with 10%.

#### 3.4.2. Compressive Strength

**Figure 15:**
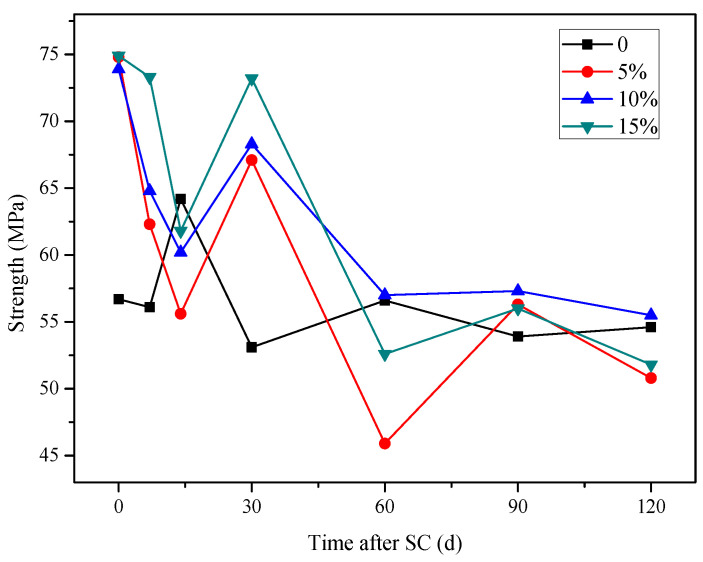
Strength of specimens with different silica fume content.

According to Figure 15, with the increase in the electrification duration, the compressive strength of the specimen with each content fluctuates, but the overall trend decreases.

The erosion of the stray current is strong in the first 15 days. A large number of C–H crystals are contained in the cement-based material and not consumed. Under the action of directional current, calcium ions move to the cathode. The strength growth provided by cement hydration and silica fume is not enough to support the erosion damage under stray current, so the strength of the first stage is reduced. In the second stage, compressive strength shows an upward trend because the accelerated dissolution of stray current has stabilized, and the internal Ca^2+^ concentration is close to the threshold. The directional migration rate of Ca^2+^ under the action of current slows down. At this time, the hydration degree of cement-based materials increases, and more dense gel phases can resist the stray current corrosion. In the third stage, the compressive strength decreases significantly. It is considered that there are two reasons: the first is that, under the continuous action of stray current, the OH^-^ in 5% NaOH solution is consumed, and the solution changes to acid, which produces a certain acid corrosion on cement-based materials, resulting in the decrease of strength. The second is that C–S–H hydrated gel, generated from the hydration of silica fume and C–H crystal, will decompose to maintain the internal charge balance of the specimen when the calcium concentration is low, resulting in structural damage and strength degradation.

After stray current is applied for 120 d, the compressive strength of the specimen increases by 1.6%, with a silica fume content of 10%, while that of the specimens with silica fume contents of 5% and 15% decrease by 7.0% and 5.1%, respectively, compared with that of the specimen without silica fume; the compressive strength of the specimens with 5%, 10%, and 15% silica fume content decrease by 32.1%, 24.9%, and 30.8%, respectively, compared with the initial strength of the specimen with each content before the stray current test. Therefore, in terms of compressive strength, the specimen with 10% silica fume content possesses the best anti-corrosion effect under stray current.

#### 3.4.3. Ca^2+^ Concentration in the Cathode Side Solution

**Figure 16:**
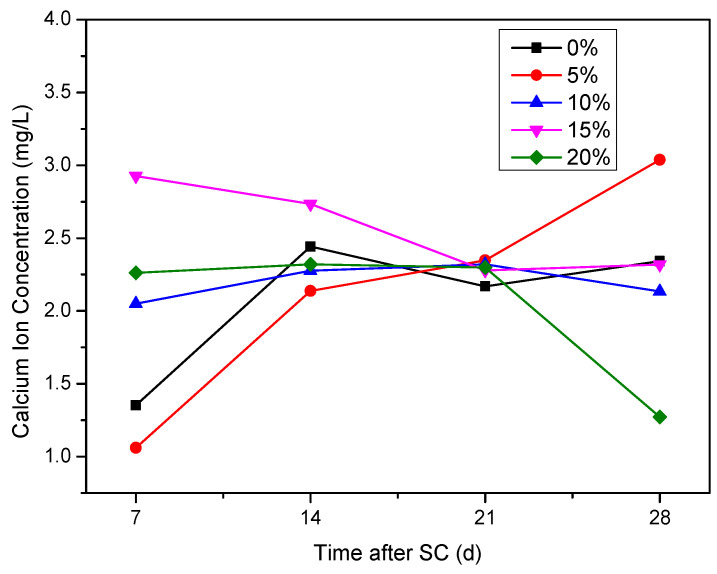
Ca^2+^ concentration in the cathode side solution of the specimens with different silica fume content.

It can be seen from Figure 16 that the Ca^2+^ concentration in the cathode side solution of the specimen generally increases during the first 14 days of stray current. At 7 d of stray current, the Ca^2+^ concentration in the cathode side solution of the specimen with 15% silica fume content is the highest of 2.93 mg/L, and that with 5% silica fume content is the lowest of 1.06 mg/L; at 28 d, the Ca^2+^ concentration in the cathode side solution of the specimen with 5% silica fume content is the highest of 3.04 mg/L, and that with 20% silica fume content is the lowest of 1.27 mg/L.

## 4. Conclusions

In this study, the corrosion of cement-based materials under the action of stray current is investigated, and the effects of different stray current magnitudes, water-binder ratios, fly ash admixtures, and silica fume admixtures on the corrosion of cement-based materials are analyzed. The main conclusions are as follows:

(1) As the stray current energisation duration increases, the mass of cathode side leachables increase and compressive strength of cement-based materials decreases overall. The mass of cathode side leachables varies with voltage as 20 < 60 < 40 V. When the voltage is 60 V, the compressive strength of cement-based materials decreases the most, which is 13.7% lower than that of specimens corroded under 0 V.
(2) At 120 d of stray current, the strength of the specimen with a water-binder ratio of 0.50 decreases the least, by only 3.7%; the mass of cathode side leachables (CaCO_3_) of the specimen with a water-binder ratio of 0.40 is the highest, and that with a water-binder ratio of 0.60 is the lowest, which is 34.52% lower than that of 0.40. Additionally, the Ca^2+^ concentration in the cathodic side solution of the cement-based materials with a water-binder ratio of 0.60 is generally the lowest, in comparison.
(3) The mass of cathode side leachables decrease significantly with the increase in fly ash content. When the content of fly ash is 15%, the mass is the lowest. In stray current environments, the compressive strength of the fly ash-doped, cement-based materials decreases more than that of the un-doped materials. At 120 d of stray current, the cement-base materials with 15% fly ash content show the smallest reduction in strength. At 28 d of stray current, the Ca^2+^ concentrations of the fly ash-doped, cement-based materials are all lower than that of the un-doped materials, and the lowest concentration is found at 10% fly ash content.
(4) When the content of silica fume is 10%, the mass of cathode side leachables of the cement-based materials is the least. At 120 d of stray current, the compressive strength of the cement-based materials with 10% silica fume content shows the least decrease, 24.9%, compared with the initial strength before the test. Therefore, in terms of the cathode side leachables and compressive strength, the specimen with 10% silica fume content possesses the best anti-corrosion effect under stray current. At 28 d of stray current, the Ca^2+^ concentration in the cathode side solution of cement-based materials with 20% silica fume content is the lowest.

In the actual subway engineering, the composition of the stray current (the respective proportion of DC and AC), direction, and periodicity are uncertain. On the basis of this study, future research can focus on effects of these practical factors.

## Figures and Tables

**Table 1:** Chemical analysis results and mineral composition of reference cement clinker (%).

SiO_2_	Al_2_O_3_	Fe_2_O_3_	CaO	MgO	SO_3_	Na_2_O_eq_	f-CaO	C_3_S	C_3_S	C_3_A	C_4_AF
22.35	4.61	3.62	65.74	2.08	0.32	0.512	0.94	56.93	21.15	6.10	11.00

**Table 2:** Chemical analysis results of cement (%).

SiO_2_	Al_2_O_3_	Fe_2_O_3_	CaO	MgO	SO_3_	Na_2_O_eq_	f-CaO	Loss	Cl
22.89	4.51	3.51	62.85	2.18	2.42	0.55	0.86	0.98	0.014

**Table 3:** Physical properties of cement.

Finesse	Density	Specific Surface Area	Standard Consistency	Stability	Setting Time (min)	
0.08/%	(g/cm^3^)	m^2^/kg	%	/mm	Initial setting	Final setting
0.9	3.15	340	25.4	0.1	193	249

**Table 4:** Component of fly ash (%).

SiO_2_	Al_2_O_3_	Fe_2_O_3_	CaO	K_2_O	TiO_2_	MgO	SO_3_	Na_2_O
55.2252	22.3812	9.1400	4.9349	3.0482	1.5964	1.2314	0.9995	0.4260

**Table 5:** Component of silica fume (%).

MgO	SiO_2_	CaO	Fe_2_O_3_	Na_2_O	K_2_O	Al_2_O_3_
0.8	96.3	0.7	0.1	0.1	1.2	0.2

**Table 6:** Mix ratio design.

No.	W/B	Water/g	Cement/g	Sand/g	Fly Ash		Silica Fume	
					Mass/g	Content	Mass/g	Content
1	0.40	180	450	1350	0	0%	0	0%
2	0.50	225	450	1350	0		0	
3	0.60	270	450	1350	0		0	
4	0.50	225	427.5	1350	22.5	5%	0	0%
5	0.50	225	405	1350	45	10%	0	
6	0.50	225	382.5	1350	67.5	15%	0	
7	0.50	225	427.5	1350	0	0%	22.5	5%
8	0.50	225	405	1350	0		45	10%
9	0.50	225	382.5	1350	0		67.5	15%

## Data Availability

The data are not to be shared.
